# Rett syndrome – biological pathways leading from MECP2 to disorder phenotypes

**DOI:** 10.1186/s13023-016-0545-5

**Published:** 2016-11-25

**Authors:** Friederike Ehrhart, Susan L. M. Coort, Elisa Cirillo, Eric Smeets, Chris T. Evelo, Leopold M. G. Curfs

**Affiliations:** 1 Governor Kremers Centre – Rett Expertise Centre, Maastricht University Medical Center, Maastricht, The Netherlands; 2 Department of Bioinformatics, NUTRIM School of Nutrition and Translational Research in Metabolism, Maastricht University, Maastricht, The Netherlands

**Keywords:** Rett syndrome, MECP2, Systems biology, Bioinformatics, Data integration, DNA methylation, Epigenetics

## Abstract

Rett syndrome (RTT) is a rare disease but still one of the most abundant causes for intellectual disability in females. Typical symptoms are onset at month 6–18 after normal pre- and postnatal development, loss of acquired skills and severe intellectual disability. The type and severity of symptoms are individually highly different. A single mutation in one gene, coding for methyl-CpG-binding protein 2 (MECP2), is responsible for the disease. The most important action of MECP2 is regulating epigenetic imprinting and chromatin condensation, but MECP2 influences many different biological pathways on multiple levels although the molecular pathways from gene to phenotype are currently not fully understood. In this review the known changes in metabolite levels, gene expression and biological pathways in RTT are summarized, discussed how they are leading to some characteristic RTT phenotypes and therefore the gaps of knowledge are identified. Namely, which phenotypes have currently no mechanistic explanation leading back to MECP2 related pathways? As a result of this review the visualization of the biologic pathways showing MECP2 up- and downstream regulation was developed and published on WikiPathways which will serve as template for future omics data driven research. This pathway driven approach may serve as a use case for other rare diseases, too.

## Background

Rett syndrome (RTT; MIM:312750) occurs in 1:10.000 girls at the age of 12 [1]. It is considered a rare disease since it affects fewer than 1 in 2000 individuals [2], but it is still one of the most abundant causes for intellectual disability in females. RTT was first described in 1966 by the Viennese pediatric Andreas Rett, who observed the typical hand movements (“hand washing”) of his patients [3, 4]. Cause of RTT is in most cases a *de novo* mutation of *MECP2* (methyl-CpG-binding protein 2) gene; which was discovered by Amir et al. [5]. However, as stated by Neul et al., “not all mutations in *MECP2* cause RTT and not all RTT patients have mutated *MECP2*”. Some *MECP2* mutations cause not RTT but a mild intellectual disability [6] and mutations in two other genes can cause a RTT like phenotype, i.e. *FOXG1* and *CDKL5*. These phenotypes were formerly considered as RTT but are now defined as RTT like syndrome [7].

RTT was considered a neurodevelopmental disorder but since some of the main symptoms were found to be reversible [8] researchers and clinicians tend to categorize it as a neurological disorder now [9]. RTT was also classified as an autism spectrum disorder as patients often develop autistic features like social withdrawal but only during a certain stage of development [10]. Although RTT has some autistic features/phases these usually disappear with time and adult RTT females are quite socially active again [11]. *MECP2* mutations are rarely found in autism patients and if, they are termed “Autism with MECP2 mutation” [7].

Recent research was able to find a correlation between certain *MECP2* mutations (or *MECP2* variants) and some phenotypes, e.g. cardiorespiratory phenotype [12], but most of the biological pathways between gene and phenotype are not yet fully understood. Especially the molecular pathways leading from *MECP2* gene to scoliosis, epilepsy or decreased growth are currently not known. In this review, we summarize the knowledge about the molecular interactions of *MECP2* gene and protein, their known downstream effects and discuss how pathway and omics data based research can elucidate the pathways towards RTT phenotypes. This review integrates database knowledge from Ensembl [13], OMIM [14], UniProt [15], The Human Protein Atlas [16], and Gene Ontology [17] and a biologic pathway was developed and published on WikiPathways [18] to visualize the mechanistic action of MECP2 and serve as a template for future omics data analysis also in other rare disease models.

## Rett syndrome phenotype development within life

Typical development of RTT starts with an “asymptomatic” first stage followed by decreased, arrested and retarded development of motor and communication skills after 6–18 months of normal postnatal development, development of stereotypic movements and loss of purposeful movement. Although the onset of typical disorder symptoms after the age of 6 months is characteristic for RTT, observations of parents that “something is wrong with this child”, are often made before. This matches with newer research which indicates that severe changes in neuronal development are apparent already at this age but due to their mild and uncertain symptoms not able to be diagnosed [19]. After a stagnation stage (2 – 10 years) which can last for years and can include some recovery and secondary gain of abilities the fourth stage typically reduces again mobility (by abnormal muscle tonus and scoliosis) while communication and cognition is preserved. RTT females are typically severely intellectual disabled, have microcephaly and seizures. Additionally, they often develop symptoms like cardiac and breathing abnormalities, gastro-intestinal problems like constipation, low muscle tension, autistic like behavior, scoliosis (and other osteopathies), sleeping problems and hormone disequilibrium. In summary, MECP2 affects epigenetic regulation of gene expression, which changes neurobiological activity, network formation and function, which causes the major phenotype. In summary, MECP2 affects epigenetic regulation of gene expression, which changes neurobiological activity, network formation and function, which causes the major phenotype. Recent longitudinal studies on the lifelong development of RTT stated that survival at the age of 25 years was 77.6% and 59.8% at 37 years [20]. The most abundant causes of death were lower respiratory tract infection, aspiration/asphyxication and respiratory failure. Two-thirds of RTT females had seizures on some point in their lives of which 36.1% were drug resistant [20]. About half of the females completely lost the ability to walk while independent walking was preserved in 17.8%. Scoliosis (85.5%) and abnormal breathing patterns (up to 88.7%) were very abundant [20]. Typical noticeable laboratory results are EEG (and EKG) abnormalities, atypical brain glycolipids, altered neurotransmitter, creatine and growth factor levels, and alkalosis. Most of the symptoms can be related to disturbed neuronal function but some of them are caused or influenced by alterations which are not yet elucidated [4, 21, 22]. It is also unknown whether it is the often in RTT observed changes of carbon dioxide metabolism that cause respiratory problems or that dysfunctional brain stem neurons are responsible for breathing abnormality [4].

## MECP2 gene, transcript and protein

**Fig. 1:**
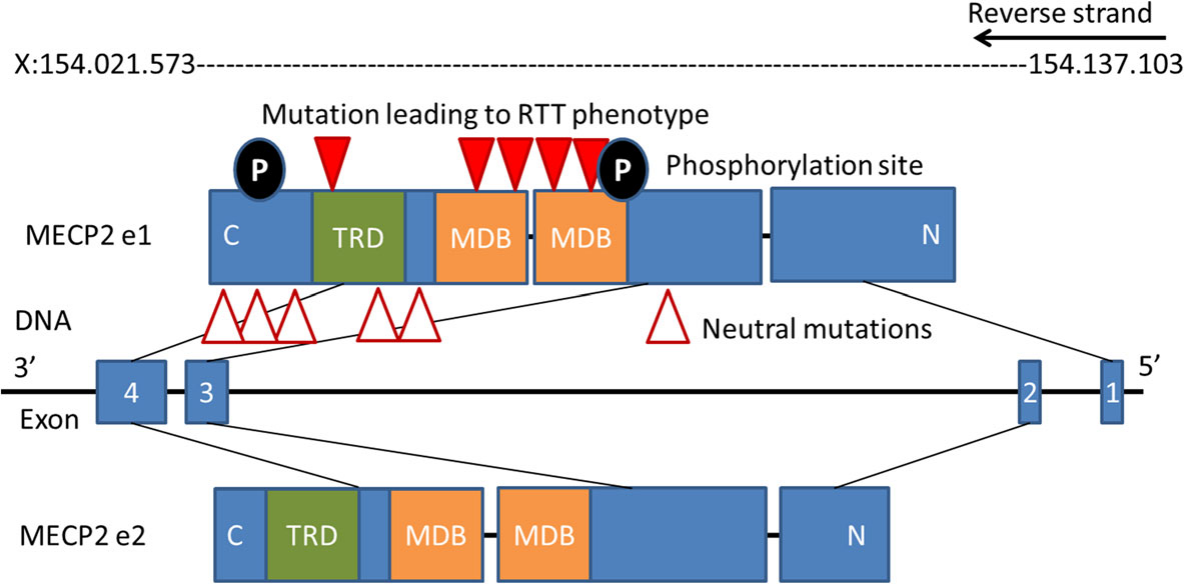
MECP2 gene and protein. *MECP2* is located on the X chromosome (X:154021573–154137103) on the reverse strand (ensembl, human genome built 8.2). *MECP2* gene is about 10505 bp long and has 4 exons which can be spliced to two protein-coding variants e1 and e2. The protein has 498 (486) amino acids and consists of 6 distinct domains whereas the methyl-DNA binding (MDB) and the transcriptional repression domain (TRD) are the most important for function. Mutation positions are marked with red arrows according to Lyst et al. 2013 [57]. Solid red arrows indicate position of mutations of MECP2, which are present in Rett females but not in their parents. Those are found mostly in MDB and the C-terminal end of TRD. Empty red arrows indicate MECP2 mutations, which do not lead to Rett syndrome. The major phorphorylation sites (S80 and S241) are marked in black [118]

The *MECP2* gene is highly conserved in Euteleostomi (bony vertebrates). The NCBI HomoloGene/UniGene database gives detailed information about gene homologues in 10 mammalian, 2 amphibian and 1 bony fish species [23]. The human *MECP2* is located on chromosome X, position 154,021,573-154,137,103 (reverse strand) (according to Ensembl, version 84, genome build GRCh38.p5 (GCA_000001405.20)) and there are currently 21 transcripts known, two of these are protein coding (Fig. 1). Due to dosage compensation *MECP2* is inactivated in one X-chromosome in females and the degree of inactivation is assumed to contribute to the difference in phenotypes for RTT [24]. RTT is more often observed in females due to its location on the X-chromosome. Hemizygous males with a severe mutation are generally not viable, but there are several non-lethal mutations which can lead to severe congenital encephalopathy, RTT-like syndrome, and mild to severe intellectual disability in males [25]. Mosaic expression with only wild-type *MECP2* active in females are possible but supposed to be extremely rare [24].

**Fig. 2:**
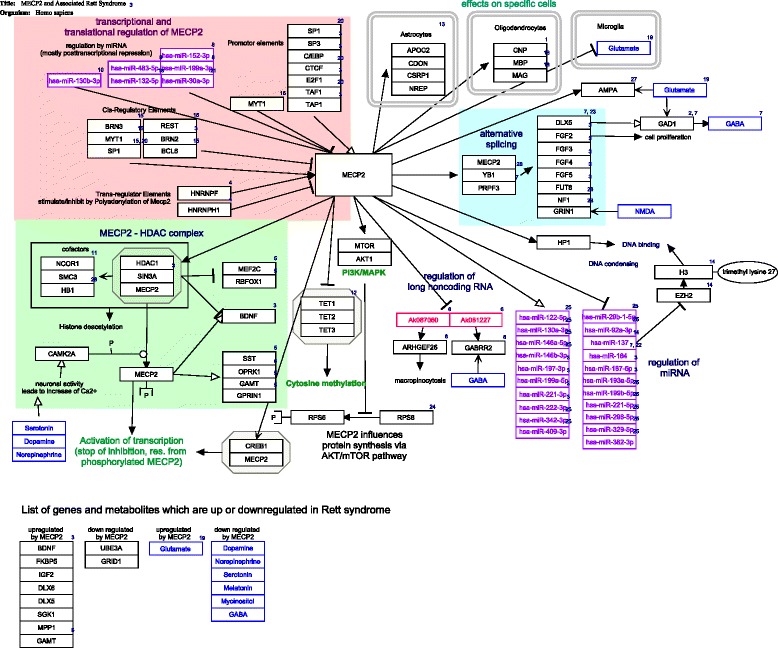
MECP2 pathway from WikiPathways (wikipathways.org/instance/WP3584). Visualization of MECP2 is regulated by several upstream elements, e.g. promotor elements and microRNAs, and MECP2 regulating the expression and splicing of several downstream transcripts, proteins and miRNAs

The transcription and translation of MECP2 is highly regulated [26] (Table 1 and Fig. 2). There are several cis- and trans-regulatory elements for *MECP2* gene expression regulation known. Cis regulatory elements, including promotor elements, are loci on the DNA which act as binding sites for transcription factors and activate or repress gene expression. Trans-elements affect the regulation in an indirect way and can be located close or far away. They can for instance include genes that encode transcription factors for this specific gene. Translation of MECP2 can be regulated by a set of microRNAs [27–35]. MicroRNAs are small non-coding RNAs, that repress translation mRNA into protein by binding to the 3’ untranslated region of the mRNA. The regulation of MECP2 expression, stimulation and repression, is visualized in Fig. 2 (transcriptional and translational regulation of MECP2) which is derived from WikiPathways [18] pathway ID 3584.

**Table 1:** Regulation of *MECP2* expression by transcription factors and microRNA

Type	Transcription factors/microRNA
Transcription factors targeting MECP2 cis-elements [27–29]	Activation by BRN3, MYT1, SP1, SP3, C/EBP, CTCF, E2F1, TAF1, TAP1
	Repression by REST, BRN2, BCL6,
Trans-regulatory elements of *MECP2* [30]	Activation by HNRNPF
	Repression by HNRNPH1
miRNA (posttranscriptional repression) [31–35]	hsa-miR-483- 5p, hsa-miR-132-5p, hsa-miR-152-3p, hsa-miR-199a-3p, hsa-miR-30a-3p, and hsa-miR-130b-3p

The two coding transcripts are isoforms of MECP2, long e1 and short e2, while e1 seems to be the more important one [36, 37] (Fig. 1). Itoh et al. observed that specific deactivation of e2 did not influence normal neurodevelopment while loss of e1 led to RTT [38]. The MECP2 protein has at least six biochemically distinct domains [39]. Two of them are most important for the protein function: the (84 amino acids) methyl-CpG binding domain (MDB) which is the one which selectively binds 5MeCyt and the transcriptional repression domain (TRD) (102 amino acids) which binds cofactors attracting histone deacetylase and finally leading to transcription repression as explained in the chapter 4 (Fig. 1) [39, 40]. Interestingly, MBD is the only structured domain (α-helix) while 60% of MECP2 is unstructured [39]. There are several post translational modifications of MECP2 known which contribute to its multi-functional properties, phosphorylation, acetylation, SUMOylation, and ubiquitination [41].

MECP2 protein is most abundant in brain but also enriched in lung and spleen tissue [42]. However, according to The Human Protein Atlas database MECP2 protein (and its transcript) is found in quite high amounts in almost every tissue, too. This may actually be the most underestimated part in RTT research which typically focus on neuronal development and function [16]. Many phenotypes and symptoms may be as well deriving from dysfunctional cellular regulation in other organs than central nervous system. In neurons an expression level of about 1.6x10^7^ protein copies per nucleus was estimated by Skene et al. It is about the same number as nucleosomes or 5-methyl-cytosine (5MeCyt) spots on the DNA, leading to the suggestion that every spot might be covered by one MECP2 [43].

## MECP2 function

**Fig. 3:**
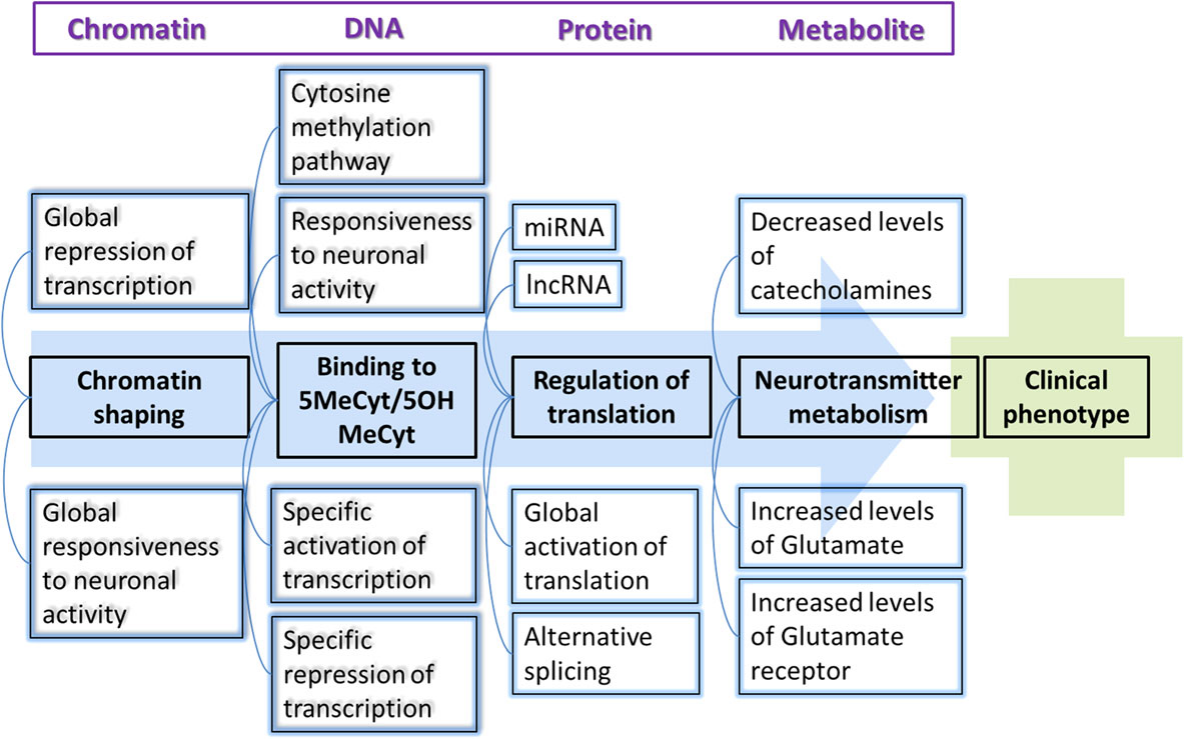
MECP2 and its different levels of influence to chromatin structure, DNA binding, protein and metabolite level leading to clinical phenotypes. 5MeCyt = methylated cytosine, 5OHMeCyt = hydroxylated cytosine

MECP2 is a multifunctional protein which influences gene expression and metabolism on many levels [9] (Fig. 3). The main function of MECP2 is to recognize and bind specifically methylated cytosine residues in the DNA (namely 5MeCyt) that are enriched with A/T bases adjacent [44]. MECP2 binds also but with lesser affinity to hydroxymethylated DNA (namely 5-hydroxy methylated cytosine, 5OHMeCyt). Mutations in MECP2, especially in the MDB, which lead to loss of specific 5MeCyt binding functions are known to cause RTT [45] (Fig. 1).

This is in line with the Gene Ontology classification for the main molecular functions of MECP2: DNA binding, namely double-stranded methylated DNA and protein binding, namely histone deacetylase. The actual full Gene Ontology annotation of MECP2 can be found online e.g. Ensembl database MECP2 entry [13].

The molecular functions of MECP2 are known to influence various biological mechanisms, which are summarized and visualized in the pathway Fig. 2, namely 1) MECP2 influences global translation by enhancing the AKT/mTOR signaling pathway [46], 2) Alternative splicing of downstream gene products is affected because MECP2 forms a complex with YB1, an important splicing factor [29, 47–51], 3) Expression of various microRNAs and long non-coding RNAs is regulated by MECP2 (20, 45, 47–49), and 4) MECP2 triggers the chromatin compaction at methylated DNA sites which regulates the transcription of adjacent genes (34, 37–41). The last one is an important (and best investigated) pathway and will be explained in detail below.

## MECP2 as epigenetic regulator

**Fig. 4:**
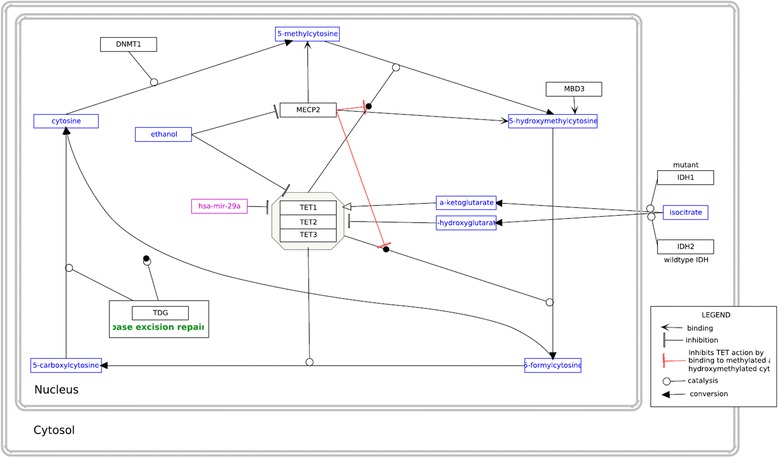
Pathway of cytosine methylation. 5MeCyt is converted to 5OHMeCyt and further to 5-formylcytosine and 5-carboxycytosine by the TET1-3 complex. MECP2 binding to these sites prevents them from being converted. The biological process is available at wikipathways.org/instance/WP3585

Methylation of DNA is part of epigenetic gene expression regulation, where DNA is modified without changing the genetic code. Most transcription factors are unable to bind to methylated DNA so methylation usually silences a gene. Furthermore, methylated DNA is – via MECP2 mediated cofactor binding - a binding site for histone deacetylase (HDAC) which increases DNA compaction by removing certain acetyl residues from lysine at the histone tail allowing them to get closer to each other (Fig. 2, MECP2-HDAC complex). So, methylated DNA is tightly wrapped around histone proteins and the access of transcription factors is physically inhibited [40]. About 1% of DNA is methylated in humans and the methylation sites are often in regions with a high occurrence of CG, so-called CpG islands. CpG islands are present in the promotor regions of most human genes (60%). Methylation patterns play a role in cellular differentiation and tissue specific gene expression already during early development [52–54]. The methylation pattern is continuously modified and maintained during mitosis and cell differentiation throughout life to grant cellular function [55, 56]. Figure 4 visualizes this circular pathway of DNA methylation, hydroxylation and de-methylation and shows the mode of action of involved proteins including MECP2.

MECP2 recognizes and binds specifically to 5MeCyt present in DNA. After binding it attracts co-repressor complexes containing SIN3A, NCOR and SMRT. This co-repressor complex finally recruits histone deacetylase (HDAC) [29, 40, 47, 57] (Fig. 2, see MECP2 – HDAC complex). The complex acts by removing certain acetyl groups from histone proteins leading to chromatin condensation around methylated DNA [58, 59]. Cell culture experiments with an inhibitor of HDAC resulted in the same phenotype as MECP2-KO cells [60]. The NCOR-SMRT interaction domain (NID) of MECP2 is located within the TRD domain and the association with NCOR-SMRT is responsible for transcription repression activity of MECP2.

MECP2 was generally considered as a transcription inhibitor but recent research found also a conditional transcription activation function. Skene et al. identified MECP2, because of its mere amount, being equal to number of histone octamers or methylated DNA sites, as a global damper of gene transcription in mature mouse brain cells [43]. During MECP2 absence H3 (histone family 3) acetylation levels were globally elevated and H1 (histone family 1) levels doubled suggesting MECP2 alters global chromatin state towards condensation and represses transcription. Li et al. on the other hand found global transcription activation by MECP2 in human embryonic stem cells which underwent differentiation to neuronal cells but repression by MECP2 in mature neuronal cells indicating that MECP2 can be both, an activator and repressor [61]. The activation mechanism is explained as follows: MECP2 recruits CREB1 as a cofactor to target gene promotors [62] (Fig. 2, Activation of transcription). MECP2 binding to 5OHMeCyt was even interpreted as a marker of active genes in neurons [62]. MECP2 was also found to form a TET1 containing complex which leads to 5MeCyt hydroxylation and further to demethylation of DNA, enabling transcription [63]. This mechanism was found to activate expression of downstream genes, namely BDNF, SST, OPRK1, GAMT, GPRIN1, and CREB1 [28] and is contradictory to other findings which describe MECP2 to block DNA demethylation by TET complex (Fig. 4) [64].

MECP2 is additionally responsible for neuronal activity triggered transcription. Neuronal membrane depolarization and Ca^2+^ influx leads to phosphorylation of MECP2 which makes it detach from DNA, allowing decondensation and transcription (Fig. 2) [65–69]. Specific blocking of MECP2 phosphorylation sites led to RTT like symptoms [70]. For a conclusion, MECP2 is responsible for the epigenetic regulation of gene expression, which changes neurobiological activity, network formation and function which is likely to cause a severe disorder phenotype if the protein is affected by mutation.

## Mutations of *MECP2* leading to RTT

At the moment, several hundred different mutations have been reported leading to RTT by loss or impaired function of MECP2 protein due to truncation, abnormal folding, or binding instability [25] (see also MECP2 varieties on LOVD database [71]). This contributes to the variety of RTT phenotype and symptom severity.

The effects of total absence of MECP2 protein were investigated in several model systems. Deletion of *MECP2* from glial cells had only mild phenotypic consequences [72]. In a mouse model specific deletion of *MECP2* in the forebrain caused behavioral abnormalities, limb clasping, impaired motor coordination, anxiety, and abnormal social behavior but not locomotor activity or changes in fear conditioning [73]. In another mouse model, silencing MECP2 in GABAergic neurons led to severe RTT like phenotype [74].

Sixty-seven percent of all *MECP2* mutations found in humans are in eight hot spots: R106 (corresponding RS number from dbSNP: rs28934907), R133 (rs28934904), T158 (rs28934906), R168 (rs61748427), R255 (rs61749721), R270 (rs61750240), R294 (rs61751362) and R306 (rs28935468). Most of the mutations which cause RTT occur in the MDB region of *MECP2* [70] (Fig. 1). A 100-fold reduction of binding affinity of MECP2 to methylated DNA is documented for the mutations R106W (rs28934907), R133C (rs28934904), and F155S (rs28934905) and binding affinity reduction of about 2-fold was found in mutation T158M (rs28934906) [45].

## The most important metabolites, genes and pathways affected by MECP2 in RTT

The examination and investigation of RTT females (and model systems) revealed that an impaired MECP2 influences biological pathways on many levels. Several genes have been found to be increased or decreased in expression, levels of various metabolites are changed and several biological pathways were found to be typically affected although the molecular mechanisms are not yet clear. In this chapter the main metabolites, genes and pathways which are influenced or changed by RTT are summarized and as far as known integrated in the MECP2 mechanistic pathway (Fig. 2). For getting these results, samples from human RTT females were often used but many results come from studies with Mecp2^-/y^ mice (e.g. the Bird model [75]). These mice do not express Mecp2 at all and they display the same symptoms as humans, such as normal development until about 6 weeks, regression, reduced movement, clumsy gait, irregular breathing, and the mice have a reduced life span of about 3 months. Postmortem analysis revealed reduced brain and neuronal cell size which is similar to observations in humans. Other mouse strains or in vitro models with mutated MECP2, reduced or overexpressed MECP2 levels are also commonly used to study RTT. Recently, researchers started to use iPSCs (induced pluripotent stem cells) from human or murine origin [76].

### Metabolites

Early autopsies revealed reduced levels of catecholamines, namely dopamine, serotonin and norepinephrine while markers for bioaminergic metabolism in general were higher [77, 78] (summary of metabolites in Table 2 and see also the list of metabolites on WikiPathways [18] (pathway ID 3584). This was confirmed by a study of Panayotiset al. [79] who revealed time-dependent levels of dopamine, norepinephrine, serotonin, and their catabolites in the brain tissues of Mecp2^-/y^ mice. Viemari [80] et al. showed that Mecp2^-/y^ mice have a deficiency in norepinephrine and serotonin content in the medulla and a drastic reduction of medullary TH (catecholamine producing) neurons indicating that dysfunctional neuronal development may be the reason for decreased catecholamine levels. The phosphorylation of MECP2 is triggered by neuronal activity which is caused by release of neurotransmitters (Fig. 2). Hutchinson et al. [81] demonstrated that phosphorylation of MECP2 is dependent on dopamine, serotonin and norepinephrine activated pathways (Fig. 2, MECP2 phosphorylation).

**Table 2:** Metabolites in RTT

Changes in RTT	Metabolites
Increased levels [77–79]	Catabolites of catecholamine metabolism, glutamate
Decreased levels [77–80]	Dopamine, norepinephrine (noradrenaline), serotonin, melatonin, myo-inositol, phospholipids, GABA

Another metabolite shown to be present in high levels in RTT females is glutamate. Increased glutamate production (or decreased glutamate consumption) may lead to over excitation of glutamatergic neurons and can trigger increased uptake and conversion of glutamate to glutamine. Over excitation feedback may cause the downregulation of the glutamate receptor which was observed before [82]. Moreover, severe downregulation in gene expression for the glutamate D1 receptor GRID1 (GluD1) was found (Fig. 2). This receptor links post and presynaptic compartments and influences not only synapsis function but also neuronal differentiation [83]. Glutamate levels during sleep-wake cycle were also affected in Mecp2 deficient mice [84]. Additionally, BDNF, which is directly regulated by MECP2, is also known to regulate GRID1 expression [85]. Glutamate disposal is energy consumptive which may help explain the increased level of energy metabolism found in brains of Mecp2^-/y^ mice and RTT females in neuroimaging studies [86].

A metabolomics investigation found changed phospholipid profiles in Mecp2^-/y^ mice [82]. Phospholipid metabolism is directly associated with cell growth since it provides membrane material for inner and outer hull structure. Still, it remains unclear whether this is the cause or just one of the consequences of reduced neuronal cell size and network connectivity of RTT. But as MECP2 basic function is global transcription dampener in combination with activity dependent activation of transcription this suggests that reduced membrane material production is a consequence of lack of activity specific transcription activation.

### Genes/gene products

MECP2 is a global transcription and translation influencing factor but there are also single specific genes which are found to be up- or downregulated in the absence of functional MECP2. The expression of the MECP2 target genes is affected in human, mouse and in vitro models. In the absence of functional MECP2 the expression of BDNF, GAMT, DLX5, DLX6, FKBP5, SGK1, FXYD1 and MPP1 is upregulated whereas the expression of UBE3A [25] and GRID1 [83] are downregulated (Table 3 and see also the list on pathway 3584 [18]). BDNF, which shows in all investigated models the most consistent effect, is a MECP2 regulated protein and it is necessary for neuronal development and function, too (Table 3). The BDNF molecular pathway and its influence on neuronal development and function is currently the best investigated. For GAMT it is known that this gene is involved in creatine metabolic and biosynthetic process which was found to be dysregulated in some (not all) Rett females and may cause or consequence to breathing problems [4]. DLX5 and DLX6 are homeobox genes and involved in regulation of gene expression in general. DLX5 is especially responsible for ectodermal differentiation processes. The activity of both is highly regulated by methylation and for the maintenance of this methylation pattern MECP2 is required: binding sites for MECP2 have been found in both promoters. There is an ongoing discussion about DLX5 and DLX6 expression being influenced by MECP2 deficiency [87], the final argument seems that it is at least true for DLX5 [88]. FKBP5 and SGK1 were identified as potential MECP2 targets because they are involved in regulation of glucocorticoid responding gene regulation (stress response). Mecp2-0 mice showed anxiety behavior, had elevated levels of those transcripts but normal levels of glucocorticoids [89]. FXYD1 is a transmembrane modulator of Na^+^/K^+^ -ATPase activity involved in neurite outgrowth. It was found to be overexpressed in a Mecp2-0 mouse model inhibiting neuronal growth [90] and reduced arborization is generally found in RTT. MPP1 (signal transduction, neuronal homeostasis) and IGF2 (cell proliferation) were identified in a transcriptomics expression study to be strongly overexpressed in a study with human lymphoblastoid cells of Rett patients and controls [91]. MECP2 interacts with E6AP, the protein of the UBE3A gene and they are regulating the expression of several target genes [92]. GRID1 encodes for glutamate D1 receptor (GluD1) and is downregulated in the absence of functional MECP2 [83]. Glutamate receptors play a major role in neuronal signal transduction.

**Table 3:** Genes, which are up- or downregulated in human or model system without functional MECP2

Changes in RTT	Genes
Upregulated [25, 89, 90]	*BDNF*, *FKBP5*, *IGF2*, *DLX5*, *DLX6*, *SGK1*, *MPP1*, *GAMT*, *FXYD1*
Downregulated [25, 83]	*UBE3A*, *GRID1*

### Pathways

Bedogni et al. mentioned the difficulty to identify unique target pathways of MECP2 because MECP2 is both a repressor and an activator of transcription and the balancing and timing of transcription levels seems to contribute more to disorder development than activation of single pathways [70]. Several transcriptomics studies using samples from RTT females, mouse and in vitro model systems showed about 60 significantly enriched pathways, for example inflammation, MAPK signaling, ERBB signaling, neurotropin intracellular cascade, sterol biosynthesis [93], cholesterol metabolism [93], cytoskeleton formation, and apoptosis [70].

While animal and in vitro models often use *MECP2* – KO models (e.g. Mecp2^-/y^ mouse) human RTT patient derived samples often have a residual MECP2 activity due to the various mutations which impair the function rather than inhibiting the expression completely. Tanaka et al. [76] investigated gene expression profiles of iPSC lines derived from six different RTT females with different *MECP2* mutations. They created and compared expression profiles of two iPSCs cell lines derived from each of the patients, one with the X chromosome active that has the wild type *MECP2* and the other one with the chromosome with the mutated gene. The differently expressed genes and altered pathways are very different for each patient. This is an interesting result, as there are so many different *MECP2* mutations which lead to RTT. A specific mutation affects protein function differently and may trigger different pathways leading to different phenotypes. Linking genetic data to molecular analysis (transcriptome, metabolome) and phenotype will be a future challenge to elucidate the pathways of RTT.

## MECP2-related pathways involved in RTT phenotypes

In this chapter we discuss how mutated *MECP2* leads to failure in neuronal synapsis formation and function which are one of the major causes of RTT phenotype. MECP2 acts in a biological (molecular) network of constant interaction by regulating and being regulated. This complex molecular network interaction leads to effects in cellular morphology (e.g. arborization), synapsis function and neuronal network growth, development, and maintenance.

According to Lyst and Bird [9] *MECP2* mutations cause RTT by disrupting two major functions: 1. co-repressor recruitment, and 2. chromatin compaction, which are both basic molecular functions. Skene and Bedogni specified this assumption of MECP2 function as a global dampener of transcription in neurons plus the activity dependent transcription activation which leads to proper synapsis function and development [43, 70].

The differences in global gene expression of RTT and wild-type control groups are not substantial – neither in fold change nor number of genes differently expressed - indicating that more subtle dysregulation events in several pathways are responsible for RTT [62, 94–97]. MECP2 is a global repressor of transcription [43], a global activator of gene translation [61] and it reacts on neuronal pathway signals which lead to phosphorylation of MECP2 and detachment from DNA. Therefore, MECP2 dampens neuronal transcription globally and allows activity related responses which seem to be necessary for learning activity and specific synapsis formation [98]. Bedogni et al. found a direct connection between MECP2 and molecular pathways leading to cytoskeleton re-formation [70] indicating structural changes leading towards neuronal network formation. BDNF and FXYD1 seem here to be the link between MECP2 and the cellular phenotype [90]. Li et al. found in human embryonic stem cells, which are developing from stem cells to neuronal precursor cells to neurons, that in a premature state, MECP2 acts as an activator of transcription while transcription repressor activity was only found in mature neurons [61]. The levels of MECP2 start to increase postnatally and the protein is quite abundant in mature nervous systems [43, 99]. The expression of MECP2 is not uniform in different neuronal cell populations [100], parts of the brain and changes with age [101]. Mouse Mecp2-KO neuronal precursor cells are not different from wildtype ones in respect to expression patterns, proliferation, and differentiation (morphology), they change only during maturation [99]. Together with the observation that symptoms of RTT do not appear before about month 6, this led to the assumption that MECP2 has less to do with neurogenesis but more with neuronal function and maintenance, and synapsis formation and function. This may explain why the RTT phenotype becomes visible only at the quite late age of 6–18 months. In RTT females brains a decreased number of synapsis was found [102–104]. Synaptogenesis again is mostly observed in the period of RTT symptom development (month 6 – 18) which may explain the development of learning disability. In MECP2 null mouse model reduced neuronal differentiation [105] and synaptic deficits [98] were observed. Mice studies and postmortem brains of RTT females reveal alterations in neuron structures which may be due to decreased dendritic complexity because of an immature synaptic spine morphology leading to malfunction of synaptic development and plasticity [106–108]. Changed neuronal tubulin expression was found directly in the brain tissue of RTT and Angelman syndrome patients [109]. Dysfunctional MECP2 led also to changes in synaptic transmission, short and long-term synaptic plasticity, deficits in short and long term potentiation (LTP and LTD) in mice [110].

The abnormal levels of neurotransmitters and differently expressed neurotransmitter receptors lead to an imbalance between excitatory and inhibitory neuronal activity (namely imbalance of GABAergic, glutamatergic and dopaminergic neuronal pathways). Such abnormal ratio of excitation/inhibition in brain activity was also found in autistic patients before [111–113] and it is a known effect in Parkinson’s disease which shares the motoric disabilities with RTT [114]. The gene in the neurotransmitter pathway which is known to be downregulated in the absence of functional MECP2 is GRID1 which could here be the link between mutation and phenotype [83]. RTT models using murine and human induced pluripotent stem cells showed some RTT features and these symptoms were documented for MECP2 overexpression models, too, leading again to the assumption that MECP2 function is dose dependent [26]. In summary, MECP2 affects epigenetic regulation of gene expression, which changes neurobiological activity, network formation and function which causes the major phenotype.

## Current gaps in understanding RTT pathways

Although these processes could indeed explain many neuronal function related symptoms of RTT there is still lack of evidence for other phenotypes, especially those which occur in many but not in all RTT females. 1) Breathing patterns: breathing is regulated by brain stem function which gets its signals from receptors for blood pH, carbon dioxide and (to a lower amount) oxygen levels. Abnormal RTT breathing patterns could be caused by neuronal dysfunction of the brain stem or neuronal pathways but metabolic dysregulation involving abnormal creatine levels due to GAMT over/under expression is also an influencing factor [4]. GAMT is one of the MECP2 downstream activated genes. 2) Cardiac abnormalities: Heart beat is generally regulated by central nervous system but has its own nerve knots for signal production, too. Specialized neurons in the heart ensure proper electric signal transition. These might be affected by RTT directly, by brain stem function, or both. RTT patients are indeed known for higher incidence of sudden death and heart problems like tachy-, brady- and arythmia were observed before (. Furthermore, vascular dysfunctions have been found which are directly related to MECP2 dysfunction although the molecular pathway between MECP2 and effector genes is not yet elucidated [115]. 3) Digestion and nutrient uptake problems: Stomach and intestines are covered with a complex network of nerve cells. Dysfunctional nerve cells may lead to constipation and malabsorption of nutrients, e.g. Vitamin D but there may also be other nutrient processing pathways involved. 4) Tissue specific effects: Tissue specific neuronal cells are differently influenced by MECP2 mutation, e.g. olfactory sensory vs. visual system (Table 4). Recently it was found that MECP2 mutations contribute to hypersensitivity of mechanoreceptors [116] which aligns with the observation of clinicians and caregivers. It is currently unknown in which neuronal cell subpopulations MECP2 is more or less necessary for normal function although there are indications that there are differences [100].

**Table 4:** Olfactory vs. visual system: tissue specific MECP2 influence

Olfactory epithelium	Visual system
Observation: A study on olfactory bulb biopsies of RTT females revealed less olfactory receptors indicating less sensitive olfactory sense [119]. The olfactory epithelium in postnatal rodents experiences strong upregulation of MECP2 [120, 121].	Observation: Vertebrate eyes are originally specialized brain tissue. Though being expected to be subjected to MECP2 dysfunctionality symptoms the visual system, retina, visual nerve and visual cortex seem to be less affected by RTT. Patients are able to focus, blink, eye-track, and do not perform worse in visual tests than healthy population [122]. Their families report often that they are using eye contact as communication method and therefore eye tracking systems are a promising method to improve communication.
Molecular/histological data: MECP2 deficiency induces also an imbalance in glutamatergic/GABAergic innervation in the olfactory bulb. The excitation in MECP2 KO mice is reduced and there is generally an imbalance between excitatory and inhibitory pathways observed leading to premature death of olfactory neurons in RTT mice models [121]. MECP2 seems to regulate the activity dependent transcriptional responses in olfactory sensory neurons the same way as in central nervous system and model systems [123]. This cycle of neuronal activity dependent transcription activation (fast feedback loop based on Ca^2+^/calmodulin) seems to be responsible for neuronal circuitry refinement, playing a role in olfactory sensory nerve maturation and olfactory learning. MECP2 affects the expression of olfactory sensory cell adhesion molecules KIRREL2 and PCHD20 directly, KIRREL3, and CNTN4 indirectly. It represses KIRREL2 but is required for activity dependent upregulation of KIRREL2 after odor stimulation [123]. KIRREL3 is an autism related gene [124] and the family of KIRREL genes is known to be widely expressed in neuronal tissue for synaptogenesis and synaptic specificity [125].	Molecular/histological data: Jain et al. investigated ocular MECP2 expression in post mortem brains of RTT females and compared it to healthy controls. Although the RTT females show the typical severe neurological deficits their visual functions are well preserved. There were no gross or microscopic aberrations detected and no significant MECP2 level differences [101]. Another study investigating MECP2 expression levels in many neuronal and non-neuronal tissues found MECP2 to be expressed weak or moderate in the nucleoplasm of retinal cells while there were peaks of strong MECP2 presence in chromocenters [126]. Although it was shown in a previous study with MECP2 KO mice that their visual system (acuity) is affected with disorder onset [127] and visual systems also need refinement by circuits and MECP2 dependent synapse remodeling [128] this was not confirmed by the study of Song et al. [126]. Their retina samples from MECP2 deficient mice did not show any differences to control concerning immunochemical markers, cellular and histological anatomy, synapsis formation and neurotransmitters [126].
Conclusion: Data indicates that the olfactory sense is less functional in Rett females due to the strong dependency of the molecular signal processing pathways on MECP2.	Conclusion: Together with the measured visual performance in human RTT females [122] these observations indicate that MECP2 surprisingly does not play a major role in ocular function.
Gap: Why is the olfactory sensory system affected in RTT females? Is there a measurable difference in response to olfactory stimulants of Rett females and controls?	Gap: What is the mechanistic explanation of the rather unaffected visual system? Why do neuronal cells of the visual system not need MECP2 for proper function?

To understand these processes integration of different levels of biological knowledge and research results is necessary, and interactive biological pathways help to organize, analyze, and visualize existing knowledge. To integrate the information the exact mutation of MECP2 gene needs to be combined with molecular data (e.g. gene expression data, metabolomics) and a detailed description of the RTT females’ phenotype (including clinical laboratory measurements). This process will increase the understanding of the underlying pathways of the variety of RTT phenotypes. Gathering this knowledge and bringing it properly together needs collaboration of biomedical and bioinformatics researchers, physicians and patients [117]. Furthermore, knowing the essential pathways and their components which contribute to a certain phenotype or symptom may lead to the discovery of drug targets. These are not likely to be able to cure RTT itself but may help to reduce the severity of specific symptoms.

## Conclusions

The present review summarizes the current knowledge about MECP2 structure and function, how it influences levels of metabolites, gene expression and biological pathways, and tries to bridge the different types of data available to explain the development of typical RTT phenotype by visualizing in form of a pathway (Fig. 2). Although the dysregulation events in neuronal cells can be already be explained quite well, the mechanistic explanation of several additional symptoms is still missing. Integrating the knowledge about the individual mutation, molecular data and phenotype information will help to find biological pathways and therefore explanations for these symptoms. Finding the right target genes, proteins, or metabolites can build a bridge between genotype and phenotype, and possibly to drug targets and pathways like this will be a great help in visualization and analyses.

## Abbreviations

Genes: Transcripts and proteins are abbreviated according to the human genome name consortium.
RTT: Rett syndrome
5MeCyt: 5-methyl cytosine
5OHMeCyt: 5-hydroxy methyl cytosine
iPSCs: Induced pluripotent stem cells
